# High flow nasal cannula in children: a literature review

**DOI:** 10.1186/s13049-016-0278-4

**Published:** 2016-07-12

**Authors:** Ingvild Bruun Mikalsen, Peter Davis, Knut Øymar

**Affiliations:** Department of Paediatrics, Stavanger University Hospital, P.O. Box 8100, N-4068, Stavanger, Norway; Department of Clinical Science, University of Bergen, Bergen, Norway; Department of Paediatric Intensive Care, Bristol Royal Hospital for Children, Bristol, UK

**Keywords:** High flow nasal cannula, Child, Mechanisms, Flow, Pressure, Effect, Ventilation, Side effect, Tolerance

## Abstract

High flow nasal cannula (HFNC) is a relatively new non-invasive ventilation therapy that seems to be well tolerated in children. Recently a marked increase in the use of HFNC has been seen both in paediatric and adult care settings. The aim of this study was to review the current knowledge of HFNC regarding mechanisms of action, safety, clinical effects and tolerance in children beyond the newborn period.

We performed a systematic search of the databases PubMed, Medline, EMBASE and Cochrane up to 12th of May 2016. Twenty-six clinical studies including children on HFNC beyond the newborn period with various respiratory diseases hospitalised in an emergency department, paediatric intensive care unit or general ward were included. Five of these studies were interventional studies and 21 were observational studies. Thirteen studies included only children with bronchiolitis, while the other studies included children with various respiratory conditions. Studies including infants hospitalised in a neonatal ward, or adults over 18 years of age, as well as expert reviews, were not systematically evaluated, but discussed if appropriate.

The available studies suggest that HFNC is a relatively safe, well-tolerated and feasible method for delivering oxygen to children with few adverse events having been reported. Different mechanisms including washout of nasopharyngeal dead space, increased pulmonary compliance and some degree of distending airway pressure may be responsible for the effect. A positive clinical effect on various respiratory parameters has been observed and studies suggest that HFNC may reduce the work of breathing. Studies including children beyond the newborn period have found that HFNC may reduce the need of continuous positive airway pressure (CPAP) and invasive ventilation, but these studies are observational and have a low level of evidence. There are no international guidelines regarding flow rates and the optimal maximal flow for HFNC is not known, but few studies have used a flow rate higher than 10 L/min for infants.

Until more evidence from randomized studies is available, HFNC may be used as a supplementary form of respiratory support in children, but with a critical approach regarding effect and safety, particularly when operated outside of a paediatric intensive care unit.

## Background

High flow nasal cannula (HFNC) oxygen delivery, also sometimes called heated humidified high flow nasal cannula (HHHFNC), is a relatively new non-invasive ventilation therapy that seems to be well tolerated in neonates and adults with hypoxemic respiratory failure [1–3]. Before the introduction of HFNC, traditionally a maximum flow of 0.5–1 L/min for delivery of oxygen by nasal cannula was set in newborns [4, 5] and a maximum flow of 2 L/min was used for older children and adults in order to prevent drying and discomfort of the nasal mucosa and other nasal mucosal complications [6]. High flow is usually defined as flow rate ≥2 L/min, the flow rate depending on the type of cannula used, but ranging from 4 to 70 L/min [7]. Debate is ongoing as to whether HFNC may reduce the use of less tolerated and more invasive ventilator supports, such as continuous positive airway pressure (CPAP) and mechanical ventilation.

HFNC was first introduced to treat preterm infants as an alternative to CPAP [5], but recently a marked increase in the use of HFNC has been seen both in paediatric and adult care settings [7–11]. In children, its use has particularly proliferated for infants and young children hospitalised with bronchiolitis. However, the evidence for the safety or effectiveness of HFNC as a respiratory support in children is relatively lacking, as underlined in two Cochrane reviews from 2014 [7, 12]. Despite that, HFNC has been increasingly implemented in clinical practice, and given that modification, it is essential that physicians should keep abreast of the latest knowledge. The aim of this study was to review the current evidence of HFNC regarding mechanisms of action, safety, clinical effects and tolerance in children beyond the newborn period.

## Methods - literature search

We performed a systematic literature search of the databases PubMed, Medline, EMBASE and Cochrane up to 12th of May 2016. We first searched for all articles with the keywords high flow nasal cannula or HFNC and limited the search to articles in English or a Scandinavian language and articles including children 0–18 years of age. The further inclusion criteria were: Studies including children with various respiratory diseases treated with HFNC hospitalised in an emergency department, paediatric intensive care unit or general paediatric ward studying mechanism of action, pressure, flow rate, clinical effect (ventilation, admission to paediatric intensive care unit, length of stay), patient comfort, safety and studies comparing HFNC to CPAP. All original clinical studies, both interventional randomized controlled studies and observational retrospective and prospective studies including children on HFNC beyond the newborn period were included and evaluated, but individual studies were not systematically assessed for the risk of bias. Details regarding study design, flow rate, outcome and key results of these studies were summarized.

From the original search, we excluded studies that did not meet the inclusion criteria in a hierarchical manner according to the following criteria.

1. Studies including only infants hospitalized in a neonatal care unit
2. Studies not corresponding to the inclusion criteria
3. Not a clinical trial
4. Studies including only adults >18 years of age

First the title of a study, as it appeared from the search was read and searched for the exclusion criteria described above. If a study could not be excluded based on the title, the abstract was read. Based on the abstract, we excluded studies that did not meet the inclusion criteria. If exclusion could not be done based on the abstract, the entire article was read.

**Fig. 1:**
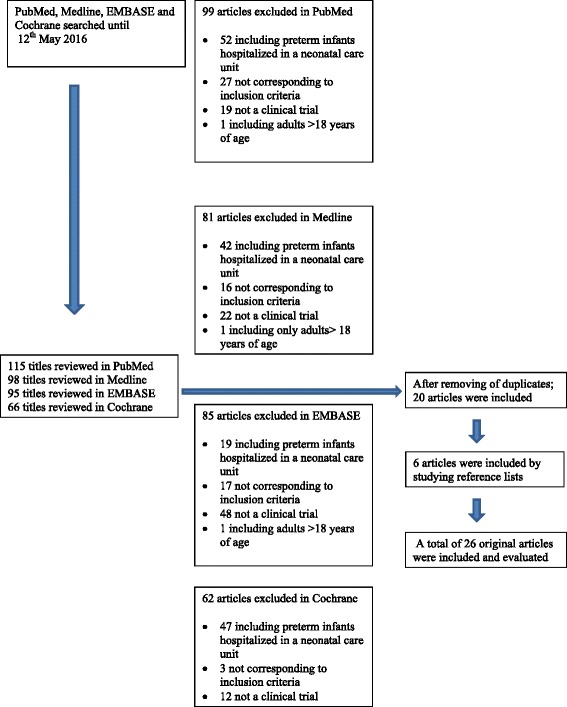
Flow diagram of the search history and the numbers of excluded and included studies

Studies including infants hospitalised in a neonatal ward or adults over 18 years of age, as well as expert reviews and Cochrane reviews, were not evaluated, but were discussed if appropriate (Fig. 1)

.

## Definition of HFNC

In the Cochrane review from 2014, HHHFNC in children was defined as heated, humidified and blended air/oxygen delivered via nasal cannula at different flow rates ≥ 2 L/min, delivering both high concentrations of oxygen and potentially continuous distending pressure [7].

## Description of clinical studies on HFNC

**Fig. 2:**
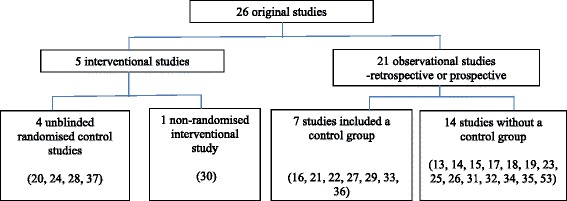
Overview of the study design of the clinical studies included in the present paper

Twenty-six clinical studies including children on HFNC beyond the newborn period were found (Fig. 2). An overview of the study design, outcome and key results of the included studies is given in Table 1.

**Table 1:** Overview of the 26 original clinical studies including children on HFNC beyond the newborn period

Author Year Citation	Study design	Study group Number of participants Age	Flow rate	Main outcomes	Key results
Children hospitalised with bronchiolitis in a general paediatric ward or emergency department					
Bressan 2013 [18]	Prospective observational.	27 infants with bronchiolitis in a general paediatric ward. Age <12 months.	Max 8 L/min.	Clinical parameters (end tidal Co2, respiratory rate, heart rate, SpO2). Feasibility of HFNC (adverse events).	Decrease in median end tidal CO2 (6–8 mmHg) and respiratory rate (13–20 per minute) in the first 3 h of HFNC and remained steady thereafter. No adverse events.
Arora 2012 [14]	Prospective observational.	25 infants with bronchiolitis in an emergency department. Age <12 months.	1 L/min, increasing with 0.5 L/min until clinical improvement, max 8 L/min.	Pressure in nasopharynx at varying flow rates of HFNC.	Increasing flow rates of HFNC up to 6 L/min were associated with linear increase in nasopharyngeal pressure.
Kallappa 2014 [31]	Retrospective observational.	45 infants with bronchiolitis in a general paediatric ward. Age <15 months.	Not given.	Clinical parameters (heart rate, respiratory rate, blood gas parameters). Adverse events.	Decrease of heart rate (median 171 to 136) and respiratory rate (median 79 to 53) and improvement in Ph (median 7.32 to 7.38) and PaCO2 (median 7.7 to 6.6 kPa), within 4 h of initiating HFNC. No adverse events.
Hilliard 2012 [24]	Prospective interventional randomized, unblinded.	19 infants with bronchiolitis in a general paediatric ward. Infants were randomized to head-box oxygen (*n* = 8) or HFNC (*n* = 11). Age <12 months.	4-8 L/min.	Safety and feasibility of HFNC in infants with bronchiolitis. SpO2 8 h after randomization and other clinical parameters at intervals up to 48 h.	Median SpO2 was higher in the HFNC group at 8 and 12 h, but similar at 24 h. FiO2 was higher in the HFNC group at all three time points.
Mayfield 2014 [16]	Observational case control. Cases were identified prospectively and controls identified retrospectively.	61 infants with bronchiolitis treated with HFNC in a general paediatric ward. 33 infants with bronchiolitis treated with standard low flow oxygen. Age <12 months.	2 L/kg/min. Max 10 L/min.	Clinical data (heart rate, respiratory rate, SpO2, LOS) admission to PICU and adverse events	Nonresponders to HFNC can be identified early. Four times higher risk of admission to PICU in the standard treatment group than in the HFNC group. HFNC is safe (no adverse events).
Bueno Campãna 2014 [20]	Prospective randomized unblinded controlled.	75 infants with bronchiolitis in general paediatric ward. 32 children on HFNC and 42 children inhaling hypertonic saline Age <6 months.	Max 8 L/min.	Respiratory distress (measured by scoring system), patient comfort, LOS, admission to PICU in the two groups.	HFNC was not superior to hypertonic saline in treatment of moderate acute bronchiolitis with respect to severity and comfort scores, LOS or PICU admission rate.
Milani [33]	Prospective observational.	36 children hospitalised with bronchiolitis in an emergency department. 18 treated with HFNC, 18 with low-flow oxygen. Age < 12 months.	8 L/kg * respiratory rate *0.3.	Respiratory rate, respiratory effort, ability to feed and LOS in the two groups.	Improvements in respiratory rate, respiratory effort and ability to feed were faster in the HFNC group. The HFNC group needed oxygen for 2 days less and LOS was 3 days shorter than in the low flow oxygen group.
Children hospitalised with bronchiolitis in paediatric intensive care unit (PICU)					
Abboud 2012 [19]	Retrospective observational.	113 children hospitalized with bronchiolitis in PICU. Age ≤12 months.	3-8 L/min.	Characteristics of non-responders to HFNC measured by respiratory rate, blood gas parameters and SaO2.	Nonresponders were more hypercarbic, less tachypnic and had no change in their respiratory rate after initiation of HFNC.
Milési 2013 [13]	Prospective observational.	21 infants with RSV bronchiolitis in PICU. Age <6 months.	1-7 L/min.	Pharyngeal pressure provided by HFCNC using flow rates from 1–7 L/min and the effect of HFNC on breathing pattern and respiratory effort.	HFNC with a flow rate equal to or above 2 L/kg/min generated a clinically relevant pharyngeal pressure ≥4 cm H_2_O and improved breathing pattern.
Hough 2014 [17]	Prospective observational.	13 infants with bronchiolitis in PICU. Age <12 months.	2 and 8 L/min. Average rate 1.7 L/kg/min.	End-expiratory lung volume, continuous distending pressure and regional ventilation distribution by measuring electrical impedance tomography.	HFNC at 8 L/min increased end-expiratory lung volume and improved respiratory rate, FiO2 and SpO2 compared with standard flow of 2 L/min. No adverse events.
McKiernan 2010 [21]	Retrospective observational cohort.	115 infants with bronchiolitis admitted to PICU during two seasons. 57 children before introduction of HFNC and 58 children after implementation of HFNC. Age <24 months.	7-8 L/min.	Intubation rate in PICU after introduction of HFNC. Clinical parameters (respiratory rate, LOS).	Intubation rate decreased from 23 % (2005–2006) to 9 % (2006–2007) after introduction of HFNC in the department. After 1 h on HFNC, respiratory rate decreased (−12 breaths/min) in infants treated with HFNC. Median LOS decreased from 6–4 days after introduction of HFNC.
Metge 2014 [22]	Retrospective observational.	34 children with bronchiolitis in PICU. 19 children on CPAP (first season) and 15 children on HFNC (second season). Age <12 months.	1-3 L/kg/min, max 8 L/min.	LOS and other clinical parameters in children on CPAP and HFNC during two seasons.	No difference between the groups in length of stay, respiratory rate, PaCO2, FiO2 and duration of oxygen support.
Riese [36]	Retrospective observational.	120 infants admitted with bronchiolitis to PICU before and 170 after introduction of HFNC in a general paediatric ward. Age < 24 months.	<6 months: 2–8 L/min. 6–18 months: 4–12 L/min. 18–24 months: 8–15 L/min.	LOS, intubation rates, 30 days readmission and median hospital charges.	LOS in PICU was reduced from 4–3 days, no difference in intubation rate or readmission, the median total hospital charges was reduced.
Children hospitalised in PICU, ICU or emergency department with various respiratory distress (also congenital heart disease)					
Pham 2014 [30]	Prospective non-randomised interventional.	14 infants with bronchiolitis. 14 infants with congenital heart disease. Admitted to PICU. Age <12 months.	2 L/kg/min.	Diaphragmatic electrical activity and oesophageal pressure changes as a surrogate for work of breath in infants off then on HFNC.	The electrical activity of the diaphragm and the oesophageal pressure-swings in infants with bronchiolitis were reduced. A similar, but less prominent offload of the diaphragm was observed in the cardiac infants.
Schibler 2011 [15]	Retrospective observational cohort.	298 infants admitted to PICU, 56 % had bronchiolitis. Age <24 months.	8 L/min at initiation.	Ventilator practice in the 5-year period after the introduction of HFNC therapy. Intubation rate.	Intubation rate decreased from 37 % in 2005 to 7 % in 2009 in infants with bronchiolitis corresponding with an increase in the use of HFNC.
Wing 2012 [29]	Retrospective observational case control.	848 patients divided in 3 cohorts admitted to PICU with acute respiratory insufficiency. 24 % had bronchiolitis. Cohort 1 (*n* = 190): HFNC not available Cohort 2 (*n* = 289): HFNC available, but no guidelines. Cohort 3 (*n* = 369): HFNC and guidelines available. Age 0–18 years.	Range 2–50 L/min. Details not given.	The need of intubation and mechanical ventilation before and after the availability of HFNC.	Intubation rate decreased from 16 to 8 % after the implementation of HFNC in PICU. No significant change in mortality or median PICU length of stay.
Rubin 2014 [23]	Prospective observational cohort.	25 patients in ICU receiving HFNC or planned to be extubated to HFNC. Age < 18 years.	2-8 L/min.	Effort of breathing in children on CPAP and HFNC at different flow rates by measuring the pressure-rate product (change in pleural pressure multiplied by respiratory rate). Oesophageal pressure was used as a surrogate for pleural pressure.	Increasing flow rates (2, 5 and 8 L/min) of HFNC decreased the pressure-rate product and increased the baseline pleural pressure.
ten Brink 2013 [27]	Prospective observational.	109 children in PICU requiring respiratory support for various disease categories; 72 children on HFNC and 37 on CPAP. HFNC: median age 6 months, CPAP: median age 5 months.	2 L/kg/min.	Level of and duration of respiratory support, and other clinical data in children on HFNC and CPAP.	No significant difference in the number of children requiring a higher level of respiratory support in the two groups. ¼ of all children on HFNC required higher level of respiratory support, these had failure of normalization of heart rate and respiratory rate and not fall in FiO2 after 2 h on HFNC.
Testa 2014 [28]	Prospective interventional randomized unblinded.	89 paediatric cardiac surgical patients in PICU. Infants were randomized to conventional O2 therapy (*n* = 46) or HFNC (*n* = 43). Age <18 months.	2 L/kg/min.	Clinical characteristics and need for higher respiratory support and reintubation rate. 48 h observational time.	PaCo2 did not differ between the group with HFNC and conventional O2 therapy. PaO2 was higher in the HFNC group. No difference in reintubation rate.
Spentzas 2009 [26]	Prospective observational.	46 neonates and children treated for respiratory distress in PICU. Patients were switched from traditional oxygen therapy to HFNC. Age 0–12 years.	8-12 L/min in infants. 20–30 L/min in children.	Tolerability and effectiveness of HFNC treatment using COMFORT scale and nasopharyngeal pressure.	COMFORT score and oxygen saturation improved in children after switching to HFNC. HFNC generated a positive end expiratory pressure of 4 ± 1.99 cm H_2_O; the pressure was dependent of weight and flow rate.
Kelly 2013 [34]	Retrospective observational.	498 children admitted to paediatric emergency department with respiratory distress, 46 % had bronchiolitis. Age < 2 years.	Not given.	Clinical and patient characteristics that predicts success or failure of HFNC therapy.	Respiratory rate > 90th percentile for age, initial venous PaCO2 > 50 mmHg, and initial venous pH < 7.30 were associated with failure of HFNC therapy. A diagnosis of acute bronchiolitis was protective with respect to intubation following HFNC.
Wraight 2015 [32]	Retrospective observational.	54 children hospitalized in PICU for various respiratory disorders. 79 % with bronchiolitis. Median age 3.5 months.	2 L/kg/min.	Failure of HFNC therapy defined as the patient needing escalation of treatment to CPAP or intubation.	HFNC was successful in 78 % of patients and failed for 12 patients (7 needed CPAP and 5 were intubated). The failure rate was 50 % in children with a primary diagnosis of congenital heart disease.
Long [35]	Prospective observational.	71 children hospitalized with various respiratory distress in emergency department. Median age 9 months.	2 L/kg/min up to 10 kg, 0.5 L/kg/min thereafter.	Failure rate, predictors of failure and adverse events.	28 (39 %) children required escalation to a higher level of respiratory support. No serious adverse events in emergency department, but one child developed air leak syndrome after transfer to ICU.
Chisti [37]	Open randomised controlled.	Children with severe pneumonia; randomised to CPAP, HFNC, or low-flow oxygen. <5 years of age.	2 L/kg/min, max 12 L/min.	Treatment failure after 1 h.	Oxygen therapy delivered by CPAP improved outcomes compared to low flow-oxygen, no difference between HFNC and CPAP group.
Children hospitalised with obstructive apnoea-hypopnea syndrome					
McGinley 2009 [25]	Prospective observational.	12 children with obstructive apnoea-hypopnea syndrome in a paediatric sleep disorder centre. 10 patients had undergone CPAP titration before study start. Age 10 ± 1 year.	20 L/min.	Numbers of obstructive sleep apnoea, clinical parameters (respiratory rate, arousals).	HFNC reduced the inspiratory flow limitation and decreased respiratory rate. HFNC decreased arousals and apnoea hypopnoea index comparable to CPAP.
Joseph [53]	Retrospective observational.	5 children with obstructive sleep apnoea not tolerating CPAP. Age < 18 years.	≤10 L/min.	Change in apnoea-hypopnoea index and oxygen saturation.	Treatment with HFNC improved the apnoea-hypopnoea index and increased oxygen saturation.

*PICU* pediatrics intensive care unit, *HFNC* high flow nasal cannula, *FiO2* fraction of inspired oxygen, *SpO2* peripheral capillary oxygen saturation, *PaCo2* partial pressure of carbon dioxide, *PaO2* partial pressure of oxygen, *CPAP* continuous positive airway pressure, *LOS* length of stay, *SaO2* arterial oxygen saturation

Thirteen studies included only children hospitalized with bronchiolitis, ten studies included children hospitalized with respiratory distress due to various airway disorders, one study included paediatric cardiac surgical patients and two studies included children with obstructive apnoea-hypopnea syndrome. The bronchiolitis studies included children up to 24 months of age, while the other studies included children up to 18 years of age. Overall, the majority of children studied were below 2 years of age. Six of the bronchiolitis studies included children in a paediatric intensive care unit (PICU), five included children hospitalised in general paediatric wards and two studies included children in emergency departments. HFNC devices with flow rates ranging from 4–10 L/min were used for children younger than 24 months of age [13–24], and flows of up to 50 L/min were used in older children [25–29].

Six studies estimated distending airway pressure [13, 14, 17, 23, 26, 30], eight evaluated feasibility and safety [16, 18, 24, 26, 27, 31–33], while five studies attempted to predict non-responders to HFNC therapy [16, 19, 32, 34, 35]. Nine studies evaluated the clinical effects measured by respiratory rate, heart rate, blood gas values, SpO2 (peripheral capillary oxygen saturation), FiO2 (fraction of inspired oxygen) and length of stay (LOS) [21, 23, 24, 26, 28, 31–33, 36], while five studies had intubation as an outcome [15, 21, 29, 32, 37]. One study compared HFNC to inhalation of hypertonic saline [20] and two studies compared HFNC to CPAP [22, 27].

## Mechanisms of action of HFNC

The suggested mechanisms of actions of HFNC are:

1. Washout of nasopharyngeal dead space resulting in increased fraction of oxygen and carbon dioxide in the alveoli [38, 39],
2. Reduction of inspiratory resistance and work of breathing by providing adequate flow [30, 39],
3. Improvement of airway conductance and pulmonary compliance by reducing the effect of cold air; an in vitro study has shown that inspired gas with low humidity even for short periods may result in worsened function of human airway epithelial cells inflammatory indices [39, 40],
4. Reduction of the metabolic cost of gas conditions by providing air with 100 % relative humidity [39],
5. Providing an end-distending pressure to the lungs [13, 17, 30, 38, 39].

## Pressure generated by HFNC

The pressure delivered to the distal airway is difficult to measure. Various indirect methods are used, i.e. pressure in oesophagus [23, 30], pharynx [13], nasopharynx [14, 26, 41], electrical impedance tomography on the surface of the chest [17] or electrical activity of the diaphragm [30]. One of the first studies published on HFNC in neonates showed that a flow of 2 L/min could generate a high oesophageal pressure of up to 9.8 cm H_2_O [42]. Recent studies have suggested limited pressure delivery as measured in pharynx and oesophagus, ranging from 2–4 cm H_2_O both in children [13, 14, 26] and adults [41]. A prospective study including 25 patients below 18 years of age, found higher pleural pressure on HFNC with flows of 8 L/min compared to flows of 2 L/min [23]. Similarly in a lung model study, the positive distending pressure to the lungs increased as the flow increased from 0 L/min to 12 L/min [43]. Overall, the distending airway pressure appears to be dependent on the weight/size of the patient, flow rate, and the diameter of the nasal cannula compared to the nares, with a higher pressure being delivered when the mouth is closed [14, 42, 44, 45]. In conventional nasal CPAP, the pressure that the patient breathes is controlled via a valve providing an escape route. In HFNC there is no equivalent control valve, and the only escape routes are the leak at the nares-prong interface and via the mouth [43, 44].

## Level of flow

The optimal maximal flow for HFNC is not known. In most studies included in this paper, the flow rate used varied from 2 to 8 L/min and was adjusted individually to minimize the patients’ work of breathing and SpO2 values. In nine studies, the flow rate was estimated by the patient’s weight [16, 22, 27, 28, 30, 32, 33, 35, 37]. Six of these studies used a flow of 2 L/kg/min, with a maximum flow of 8–12 L/min being used in two studies (Table 1). One study reported a flow rate varying from 1 to 3 L/kg/min, but a max flow of 8 L/min [22]. In a study including children hospitalized with bronchiolitis in a general paediatric ward, a flow of 2 L/kg/min, with a max flow of 10 L/min was safe with no adverse events [16].

In a recently published review from Hutchings et al., a guideline for the initiation and strategies for escalation and weaning of HFNC in a general paediatric ward was suggested [46]. In this local guideline, the initial flow is set dependent on age, and the flow is increased if the points in a particular patient scoring system are above a given trigger level. The authors discuss the alternative of using flow rates per kg, but underline that such an approach might result in very high flow rates.

As shown in the present paper, few studies including infants have used a flow rate above 10 L/min, and there are no studies comparing flow rates above 10 L/min and pressure. Higher flow rates up to 50 L/min have been used in studies including older children and adults [26, 29, 47]. Flow rates up to 1.5-2 L/kg/min are being used in children both in general paediatric wards and PICUs (Table 1). However, the lack of studies using higher flow rates and the few case reports of serious air leakage in children treated with HFNC [48] indicate that caution should be exercised with increasing flow rates higher than 1 L/kg/min in children or higher than 10 L/min for infants, particularly outside of a PICU.

## Clinical effects

### Ventilation and oxygenation

In a prospective randomized open pilot study including 19 infants hospitalised with bronchiolitis, a higher median SpO2 at 8 and 12 h, but not at 24 h, was found in the HFNC group than in a group receiving head-box oxygen [24]. In a RCT of children undergoing cardiac surgery, improvement of partial pressure of oxygen/fraction of inspired oxygen (PaO2/FiO2) was found after extubation in children receiving HFNC compared to oxygen given by cannulas with a maximum flow rate of 2 L/min [28]. A reduction in respiratory rate and improvement of blood gas parameters has also been reported in other prospective bronchiolitis studies, details are given in Table 1 [18, 21, 26, 31, 33].

### Admission to PICU and length of stay

The only case control study on the effect of HFNC on admission to PICU found that admission was four times less likely in children receiving HFNC than children receiving standard treatment [16]. However, there was no difference in the length of stay (LOS). One small prospective observational study including children with bronchiolitis found that LOS was 3 days shorter in children receiving HFNC than children receiving low flow oxygen [33]. Another retrospective bronchiolitis study found that the median hospital LOS was 4 days vs. 3 days before and after the introduction of HFNC in the general wards [36]. However, no differences in LOS were found in a study comparing children with bronchiolitis treated with HFNC and hypertonic saline [20], or in a bronchiolitis study comparing children on CPAP and HFNC during two seasons [22]. Similarly there were no differences in LOS in an RCT comparing children undergoing cardiac surgery with conventional oxygen therapy and HFNC [28], or in a retrospective observational case control including children aged 0–18 years admitted to PICU with acute respiratory insufficiency due to various respiratory diseases [29]. The median LOS in PICU was reduced from six to four hours in children hospitalised with bronchiolitis treated with HFNC compared to children hospitalized in seasons before the introduction of HFNC [21], but this finding probably has limited clinical importance, given the very short LOS reported.

In summary, studies on the effect of HFNC have identified a positive clinical effect on SpO2, PaO2, respiratory rate and blood gas parameters in some children, especially for children with bronchiolitis. In children with bronchiolitis, also some effect of HFNC has been found on LOS and admission to PICU, but not in children with other respiratory diseases.

## Patient comfort with high flow

Only one small study in children outside the neonatal period has studied patient tolerance and compliance. This study included 46 children with various causes of respiratory distresses from 0 to 12 years of age, and found that patient comfort measured by COMFORT scale improved when switching from oxygen delivered by nasal cannula or face mask to HFNC [26]. In a small study including 20 adults, high flow was reported to be more comfortable and associated with less dyspnoea and mouth dryness compared to oxygen delivered via face mask [3]. In a Norwegian study among newborns, no difference was found in patient comfort on HFNC and CPAP, but parents preferred HFNC to CPAP, reporting that their child was more satisfied, and that they perceived that it was easier to interact with their child when they were on HFNC [1]. However, a study on preterm infants found no difference in noise levels between CPAP and HFNC [2]. The results of these studies in neonates may also be valid for young infants hospitalised with bronchiolitis.

A survey from Australia and New Zealand directed at senior medical and nursing staff noted that, despite a lack of guidelines, HFNC was perceived as easy to administer and comfortable for infants [11]. It would seem that this assessment of improved patient tolerance when using HFNC compared to other forms of respiratory support may also help explain its popularity with clinical staff, and would appear to be one of the reasons for its increasing use over recent years, despite a lack of evidence for its clinical effectiveness.

## Identification of non-responders

One study including children hospitalised with bronchiolitis, identified responders and non-responders to HFNC within 60 min of treatment; responders had lower heart and respiratory rates, whereas no equivalent changes were found among non-responders [16]. Similarly, early identification of non-responders was found in children on HFNC hospitalised in a PICU for various causes of respiratory distress, with a median increase in respiratory rate at 1 h in the HFNC failure group [32]. Another study also looking at young children with bronchiolitis concluded that non-responders had no improvement in their respiratory rate after the initiation of HFNC, were more hypercarbic but also had a lower respiratory rate prior to the start of HFNC, suggesting that perhaps they were already tiring [19]. In a study of children under 2 years of age presenting to an emergency department with respiratory distress, non-responders had a respiratory rate above the 90th percentile for age, an initial venous partial pressure of carbon dioxide (PaCO2) above 50 mmHg (6.7 kPa), and an initial venous pH less than 7.30 [34]. Measurement of a blood gas and the recognition of hypercarbia, respiratory acidosis and tachypnea, may allow for early identification of infants and children at increased risk of not responding to HFNC, and therefor may be in need of additional respiratory support.

## High flow nasal cannula compared to CPAP

There is only one randomized controlled trial comparing CPAP and HNFC in children after the newborn period [37]. This study of children with severe pneumonia in Bangladesh, found that when CPAP was compared to low flow oxygen it improved outcome (intubation, death, clinical failure), but found no difference in outcome between children supported by HFNC or CPAP. A small retrospective study comparing children on HFNC and CPAP during two seasons, found no difference between the groups regarding length of stay, respiratory rate, PaCO2, FiO2 or duration of oxygen supply [22]. Similarly, another prospective study found no significant difference between children on HFNC and CPAP regarding respiratory rate, heart rate, arterial oxygen saturation (SaO2) or respiratory distress. In this study, 26 % of the children on HFNC required an escalation of respiratory support compared to 18 % in the CPAP group (*p* = 0.27) [27].

An observational study investigating the pressure delivery system in vitro and in vivo on newborns, found similar end-expiratory oesophagus pressures for neonates treated with HFNC and CPAP [49]. In neonates and adults, randomized controlled trials have shown no different effects of CPAP and HFNC regarding intubation. In preterm babies three randomized controlled non-inferiority trials found similar effects of HFNC compared to CPAP after extubation [50–52].

## Intubation

Five retrospective observational studies have assessed the use of HFNC and the risk for intubation in children [15, 21, 29, 32, 36]. Three of these studies concluded that the use of HFNC was associated with an overall reduction in the intubation rates, however these studies had a low level of evidence [15, 21, 29]. Two of the studies on children with bronchiolitis below 24 months of age, started with a flow rate of 8 L/min [15, 21]. In the study by Wing et al., children aged 0–18 years with other conditions than bronchiolitis were included, with flows varying from 8 to 50 L/min depending on the age of the child [29]. A fourth study with intubation as outcome used a flow rate of 2 L/kg/min, but did not include a control group [32]. They reported that 12 % of infants and children hospitalised to PICU for various respiratory disorders supported on HFNC were in need of a step-up treatment with CPAP or intubation. Another study found no difference in intubation rate before and after the initiation of HFNC in a general paediatric ward [36], while in a further observational study, approximately one-third of children commenced on HFNC in an emergency department required escalation to a higher level of respiratory support (CPAP or intubation) [35]. It is also worth noting that although a recently published RCT in adults found an overall decrease in mortality on HFNC at a flow of 50 L/min compared to non-invasive ventilation, there was no overall reduction in the intubation rate when compared to standard oxygen or non-invasive ventilation [47].

## Role of high flow for other conditions than bronchiolitis

A Cochrane analysis from 2014, studying the effect of HFNC in children with other conditions than bronchiolitis, found no RCT and concluded that no evidence was available to determine the safety or effectiveness of HFNC as a form of respiratory support in children [7]. One small study has reported less effect in children with respiratory distress due to congenital heart disease than that with bronchiolitis [30]. An association between heart disease and higher failure rate of HFNC has also been observed [32]. However, in a recent published RCT studying HFNC compared to conventional oxygen therapy during the first 48 h after extubation for cardiac surgery, HFNC improved PaO2, but not PaCO2 [28]. Clinical improvement by HFNC in children with obstructive sleep apnoea has been found in two small studies [25, 53]. Case reports have also described an effect of HFNC in children with acute pulmonary oedema [54] and a paediatric burn patient with post extubation stridor [55].

## Side effects and safety

Most studies have reported no adverse events for children on HFNC and have concluded that the use of HFNC is safe both in a general paediatric ward [16, 20, 31], emergency department [14] and PICU [17, 27].

However, two reports described four serious cases of pneumothorax in children on HFNC; one 2 month old child treated for RSV bronchiolitis (flow rate 6–8 L/min), one 16 year old child with cerebral palsy (flow rate 15–20 L/min), one 22 months old boy with a subdural hematoma (flow rate 6 L/min) [48] and also in a 4 year old child with asthma treated with HFNC (flow 40 L/min) [35]. Unlike CPAP, which may be delivered by systems with an integrated pressure relief valve, it is not possible to regulate or determine the pressure applied to the airways in HFNC. In vitro and in vivo studies underline the risk of an HFNC device delivering high pressures at higher flow rates, particularly if there is minimal leak [42, 43, 45].

Three studies have reported abdominal distension in children on HFNC, indicating that one should be careful with HFNC in children with intra-abdominal pathology [27, 28, 35]. Mucosal injury with nasal bleeding and ulceration has been reported in children on HFNC [27], but in a RCT including preterm infants below 32 weeks, nasal trauma was less frequent in the HFNC group than in the CPAP group [56].

An outbreak of *Ralstonia mannitolilytica,* a waterborne opportunistic human pathogen, was found among paediatric patients receiving HFNC in the US in 2005. The outbreak was linked to intrinsic contamination of the HFNC devices [57], but since changes to the device no further infectious complications have been reported.

## Conclusion

The majority of the studies on the use of HFNC beyond the newborn period are small observational studies, with a limited level of evidence of its use in infants and young children. The results from the available studies suggest that HFNC is a relatively safe, well-tolerated and feasible method for delivering oxygen to infants and young children in a general paediatric ward. Different mechanisms including washout of nasopharyngeal dead space, increased pulmonary compliance have been postulated, but it is possible that some amount of distending airway pressure may be the main reason for the effect.

Most of the clinical studies in children have been observational studies conducted in infants with bronchiolitis. A positive clinical effect on various respiratory parameters has been detected, and studies suggest that HFNC may reduce the work of breathing. HFNC may also decrease the need of CPAP and invasive ventilation in infants and children. RCTs performed in preterm infants and adults suggest that HFNC may be as effective as CPAP following extubation, while in children who have undergone cardiac surgery it has been found to improve oxygenation in the post-extubation period, when compared to low flow oxygen.

There are no international guidelines regarding flow rates, and the varying flow rates used in the clinical studies described in this paper, may explain the different results regarding effect. RCTs of HFNC including children beyond the newborn period are currently ongoing [58]. Until more evidence is available, HFNC may be used as a supplementary form of respiratory support in infants and children, but with a critical approach regarding effective clinical responses and safety issues relating to early recognition of treatment failure, particularly when children are managed on HFNC outside of a paediatric intensive care unit.
